# Effects of Lower Limb Length and Body Proportions on the Energy Cost of Overground Walking in Older Persons

**DOI:** 10.1155/2014/318204

**Published:** 2014-06-17

**Authors:** Federica Vannetti, Guido Pasquini, Nicola Vitiello, Raffaele Molino-Lova

**Affiliations:** ^1^UO Riabilitazione Cardiaca, Fondazione Don Carlo Gnocchi, Via di Scandicci snc, 50143 Florence, Italy; ^2^Istituto di BioRobotica, Scuola Superiore Sant'Anna, Viale Rinaldo Piaggio 34, 56025 Pontedera, Pisa, Italy

## Abstract

*Background*. Although walking has been extensively investigated in its biomechanical and physiological aspects, little is known on whether lower limb length and body proportions affect the energy cost of overground walking in older persons. *Methods*. We enrolled 50 men and 12 women aged 65 years and over, mean 69.1 ± SD 5.4, who at the end of their cardiac rehabilitation program performed the six-minute walk test while wearing a portable device for direct calorimetry and who walked a distance comparable to that of nondisabled community-dwelling older persons. *Results*. In the multivariable regression model (*F* = 12.75, P < 0.001, adjusted R^2^ = 0.278) the energy cost of overground walking, expressed as the net energy expenditure, in kg^−1^ sec^−1^, needed to provide own body mass with 1 joule kinetic energy, was inversely related to lower limb length and directly related to lower limb length to height ratio (*β* ± SE(*β*) = −3.72∗10^−3^ ± 0.74∗10^−3^, *P* < 0.001, and 6.61∗10^−3^ ± 2.14∗10^−3^, *P* = 0.003, resp.). Ancillary analyses also showed that, altogether, 1 cm increase in lower limb length reduced the energy cost of overground walking by 2.57% (95%CI 2.35–2.79). *Conclusions*. Lower limb length and body proportions actually affect the energy cost of overground walking in older persons.

## 1. Introduction

The transition from quadrupedal knuckle walking to striding bipedalism was a distinctive evolutionary event that marked the divergence of hominin lineage from other apes [1, 2]. This critical transition occurred in the late Miocene (8 to 5 Million years ago) when the climate became cooler and drier and the lush subtropical forests were replaced by savannahs [3]. As the “Miocene Disruption” considerably increased the distance between food patches, in 1980 Rodman and McHenry [4] first hypothesized that this transition evolved in early hominins to improve walking metabolic efficiency, thus providing our ancestors with the evolutionary advantage over other apes of reducing the energy cost of foraging. Interestingly, more recent studies, by applying biomechanical models and gas exchange measurements, have shown that the energy cost of bipedal walking in A.L. 288* Australopithecus afarensis* specimen over a wide range of postures, from crouched to fully extended, along with being actually lower than that of quadrupedal apes, was also higher than that of contemporary humans and that this difference was accounted for by the difference in lower limb length, with contemporary humans' longer limbs resulting in more efficient walking [5–7].

Independent of foraging efficiency, which has been progressively losing its relevance over time, walking still continues to be an important function in contemporary humans. It is critical to maintain independence in activities of daily living, to enjoy social relationships, and to retain good emotional vitality, all of which are main determinants of quality of life, particularly in older persons [8]. Further, walking also provides older persons with extensive benefits in terms of morbidity and mortality by controlling cardiovascular risk factors, by limiting the progressive loss of muscle mass and strength, by maintaining joint flexibility and bone resistance, and by stimulating more complex functions, such as balance and coordination, that are critical to prevent falls [9]. Unluckily, people walk progressively less with advancing age, even in the absence of clear-cut chronic functional impairments [8] and the habitual speed of walking also declines as a compensatory action aimed at offsetting the decline in walking metabolic efficiency [10, 11] and in cardiopulmonary capacity [12], both occurring with advancing age.

Although human walking has been extensively investigated in its multifaceted biomechanical, physiological, and pathological aspects [13, 14], little is known on whether lower limb length and body proportions affect the efficiency of overground walking in older persons. In fact, those studies that have addressed the issue [5–7] have assessed energy expenditure in contemporary children and younger adults walking on a treadmill, which has been shown to be significantly higher and not applicable to overground walking in older persons [15, 16]. Further, overground walking is a more natural task when compared to treadmill walking, particularly for older persons, and, contrary to treadmill walking, individuals self-select their own walking speed.

Understanding whether lower limb length and body proportions affect the energy cost of overground walking in older persons is a relevant issue. In fact, the ability to walk a certain distance depends on whether the amount of oxygen that can be delivered to muscles by the cardiopulmonary system meets the amount of metabolic energy required for walking, and, intuitively, for a given level of cardiopulmonary capacity, the lower the metabolic cost of walking, the longer the expected walked distance. Interestingly, findings from a recent study on younger adults walking on a treadmill using articulated stilts [17] suggest that changes in lower limb length/geometry might be an important factor in minimizing the energy expenditure of human walking. As the use of stilts cannot be proposed to older persons and neither the data observed during treadmill walking can be directly transferred to overground walking, investigating whether lower limb length also affects the energy cost of overground walking in older persons is an intriguing question as it lets us foresee the possibility of reducing the energy cost of overground walking by simply “manipulating” lower limb length/geometry, thus overcoming, at least in part, the limitations to physical performance imposed by the cardiopulmonary system. This might be particularly important for older persons and for patients with severely limited cardiopulmonary capacity, in whom a relatively small change in the energy cost of walking might dramatically improve their mobility and, consequently, their independence in the activities of daily living and their quality of life, which is a relevant target for cardiopulmonary and geriatric rehabilitation.

Thus, in this study we tested the above hypothesis in a selected sample of older patients who performed the six-minute walk test (6 mWT) at the end of their cardiac rehabilitation program while wearing a system for direct calorimetry.

## 2. Methods

### 2.1. Study Sample

Participants were enrolled among patients admitted to our rehabilitation centre for an intensive postacute three-week rehabilitation program after cardiac surgery over a six-month lapse of time. All patients aged 65 years and over who, at the end of the cardiac rehabilitation program, walked on the 6 mWT a distance included within the 95%CI of the normative data for nondisabled community-dwelling older persons according to sex and age strata [18], were included in the study. The final sample was represented by 62 patients, 50 men and 12 women, mean age 69.1 years ± SD 5.4, all of whom signed their informed consent form. The study was approved by the Institutional Review Board and involved no discomfort or inherent health risks for participants.

### 2.2. Anthropometric  Measures

Weight, in kilograms, height, in centimeters, and lower limb length (from greater trochanter to the ground), in centimeters, were measured unshod using commercially available tools. To obtain a measure of body proportions, we also calculated the lower limb length to height ratio, in percentage.

### 2.3. Assessment of Energy Expenditure during the 6 mWT

At the end of their cardiac rehabilitation program, patients performed the 6 mWT according to the recommendations of the American Thoracic Society [19] wearing standard shoes. The metabolic cost of overground walking was assessed using a portable device for direct calorimetry (SenseWear Armband, from BodyMedia Inc., Pittsburg, PA, USA). The system, by using built-in sensors, continuously gathers information on movement, number of steps, heat flux, near body temperature, skin temperature, and galvanic skin response. An algorithm developed by the manufacturer (SenseWear Professional software, release 6.1) allows the measurement of energy expenditure and number of steps. The SenseWear Armband was positioned on the right arm, over the triceps muscle halfway between the acromion and the olecranon processes, as suggested by the manufacturer, at least two hours before the test. Previous studies have confirmed the validity and reproducibility of the SenseWear Armband measures of energy expenditure [11, 20–22].

We measured the walked distance, in meters, the number of steps, the resting metabolic rate before the test, in joules min^−1^, and the cumulative gross energy expenditure throughout the test, in joules. Then, we calculated the mean walking speed (walked distance/360), in meters sec^−1^, the mean kinetic energy (1/2* mv* ^2^, where* m* is body mass and* v* is walking speed), in joules, the cumulative net energy expenditure (cumulative gross energy expenditure-resting metabolic rate times 6), in joules, and the cost of locomotion [23] (cumulative net energy expenditure/weight/360), in joules kg^−1^ sec^−1^. The ratio of the cost of locomotion to mean kinetics energy, that is, the net energy expenditure needed to provide patients' own body mass with 1 joule kinetic energy, was considered as the normalized measure of the energy cost of overground walking on the 6 mWT. Finally, we also calculated step cadence (step number/360), in sec^−1^, step length (walked distance/step number), in meters, and its reciprocal, that is, the number of steps per meter (step number/walked distance), in meters^−1^.

### 2.4. Statistics

Statistical analysis was performed using the STATA 7.0 software, from Stata Corporation (College Station, TX, USA). The association of the energy cost of overground walking with anthropometric measures was tested using a multivariable backward regression model with the energy cost of walking as the dependent variable and with lower limb length and lower limb length to height ratio as independent variables, along with age and sex. Height was not entered into the model as it was used to calculate the lower limb length to height ratio, thus introducing structural collinearity among independent variables. Weight was also not entered into the model as it was used to calculate the cost of locomotion. Type 1 error was set at the two-sided 0.05 level. Finally, as variables entered into the model were expressed in different units, standardized beta coefficients, that is, the *β*-coefficients calculated after standardizing all variables as having a mean of 0 and an SD of 1 (*Z*-scores), are also reported to assess the relative magnitude of the effect of independent variables on the dependent variable.

Ancillary analyses aimed at assessing the effects of 1 cm increase in lower limb length on the lower limb length to height ratio and on the energy cost of overground walking were conducted by creating first two dummy variables, “lower limb length plus one” and “lower limb length plus one to height plus one ratio.” Then, we calculated the energy cost of walking following 1 cm increase in lower limb length, “cost of locomotion to kinetics energy ratio plus one,” according to the multivariable regression model. Finally, we compared the measured cost of locomotion to kinetics energy ratio with the calculated “cost of locomotion to kinetics energy ratio plus one.”

## 3. Results

Table 1 shows the characteristics of the study sample and the results from the 6 mWT.  Table 2 shows the multivariable backward regression model testing the association of the energy cost of overground walking with anthropometric measures. Independent of the confounding effect of age and sex, lower limb length was inversely related to the energy cost of overground walking (the longer the lower limb, the lower the energy cost of walking) while lower limb length to height ratio was directly related (the higher the ratio, that is, the longer the lower limb with respect to height, the higher the energy cost of walking).

Ancillary analyses showed that in our series 1 cm increase in lower limb length increased lower limb length to height ratio by 0.284 (95%CI 0.279–0.288) and that, altogether, 1 cm increase in lower limb length reduced the energy cost of overground waking by 2.566% (95%CI 2.345–2.785).

## 4. Discussion

In this study we tested the hypothesis that lower limb length and body proportions might affect the energy cost of overground walking in older persons. In a sample of older patients who completed their cardiac rehabilitation program and who were functionally comparable to nondisabled community-dwelling older persons according to age and sex, we found that the energy cost of overground walking was inversely related to lower limb length and directly related to lower limb length to height ratio.

To the best of our knowledge, these findings have never been reported before, so that direct comparisons with the existing literature are not feasible. However, a few comments are warranted.

First, normalized measures of walking energy expenditure, such as the “cost of locomotion” (joules kg^−1^ sec^−1^) or the “cost of transport” (joules kg^−1^ m^−1^) [23], have been used to normalize the energy cost of treadmill walking referred to the experimentally preestablished walking speed. However, in the case of the 6 mWT, in spite of the encouragement of the patient provided by the assistant physiotherapist every minute throughout the test [19], walking speed is not experimentally preestablished but is in the control of the patient, thus introducing a further, unpredictable degree of freedom. As a consequence, to make results comparable between patients, we further normalized the cost of locomotion to the kinetic energy provided to patients' own body mass as it represents the reciprocal of efficiency, a quite familiar and intuitive concept.

Second, previous studies have already shown that lower limb length actually affects the energy cost of walking [5–7]. However, these studies were conducted in children and younger adults walking on a treadmill at preestablished speeds. Thus, our findings add the notion that lower limb length also affects the energy cost of overground walking in older persons. Understanding the reasons why lower limb length affects the energy cost of walking was beyond the scope of this study. However, in a separate analysis we found that the energy cost of overground walking in older persons was directly related to the number of steps per meter (*β* ± SE(*β*) = 0.12 ± 0.03, *P* < 0.001, adjusted *R* ^2^ = 0.209) and that the number of steps per meter was, in turn, inversely related to lower limb length (*β* ± SE(*β*) = −6.4∗10^−3^ ± 2.3∗10^−3^, *P* = 0.006, adjusted *R* ^2^ = 0.103). These findings obtained in older persons freely walking overground along a corridor somehow confirm the results from the study of Donelan et al. [24] on treadmill walking in younger adults that have shown that the mechanical work needed to redirect the center of mass from one pendular arc to the next during the transition between steps is a major determinant of the energy cost of human walking.

Third, for the first time we have shown that lower limb length to height ratio, a measure of body proportion, is also directly related to the energy cost of overground walking in older persons. Although the interpretation of this finding is, indeed, a difficult task, a possible explanation might be as follows. Lower limb to height ratio can be somehow considered as a proxy of the position of the center of mass with respect to the ground. In fact, if two individuals show the same lower limb length and different height, then the center of mass of the individual with a higher ratio of lower limb length to height, that is, the shorter individual, will be closer to the ground than that of the taller one. Interestingly, Abe et al. [25] in their experiments about the effects of load carriage on the energy cost of walking in younger adults found that the energy cost of treadmill walking was significantly lower while carrying the load on the upper back than on the lower back. To explain their findings, the authors hypothesized that the center of mass of the added load would generate a rotation torque around the body center of mass that would contribute to the forward propulsion during walking, with the higher position of the added load generating a more powerful torque. We do not completely agree with this interpretation as the complex “human body plus added load” has a single center of mass that is displaced upward and backward with respect to the original position of the body center of mass by the addition of load on the upper back. However, in analogy to Abe et al. [25], it might be reasonably hypothesized that the higher position of the center of mass in individuals with a lower ratio of lower limb length to height might generate a more favorable inertial moment with respect to the intertrochanter axis, the natural flexion axis between lower limbs and trunk, and that this might contribute to the forward propulsion during walking.

Finally, our ancillary analyses showed that in spite of the opposite effects of lower limb length and lower limb length to height ratio, the combined effect of 1 centimeter increase in lower limb length, and the consequent increase in lower limb to height ratio, altogether reduced by ~2.5% the energy cost of overground walking in older persons. This finding offers a new insight into exercise physiology as it suggests that changes of few centimeters in the distance from the great trochanter to the ground will result in appreciable reductions in the energy cost of overground walking. This might be particularly relevant for older persons and for patients with severely limited cardiopulmonary capacity, in whom a relatively small reduction of the energy cost of walking might dramatically improve their mobility and, consequently, their independence in the activities of daily living and their quality of life.

Contrary to previous studies, in which the effects of lower limb length on energy expenditure were assessed in children and younger participants walking on a treadmill at preestablished walking speeds, in this study we assessed the effects of lower limb length and lower limb length to height ratio on energy expenditure in older persons freely walking overground along a corridor at their self-selected speed, which is a more natural task and shows close similarity to the activities of daily living. This aspect represents the strength of our study. Nevertheless, some inherent limitations need to be considered. First, our sample size was relatively small (62 subjects), which might have affected the statistical power of our calculations; however, post hoc statistical power of the multivariable regression model was >0.99. Second, our multivariable regression model showed a relatively small adjusted *R* ^2^ (0.278), which represents the amount of the variance of the dependent variable (energy cost of walking) “explained” by the variance of independent variables (lower limb length and lower limb length to height ratio). This is a quite common finding in studies on older persons as several factors, such as balance, coordination, joint function, muscle strength, cardiopulmonary capacity, psychological status, and so forth, may affect physical functioning. Third, the amount of the reduction of the energy cost of walking following 1 cm increase in lower limb length, as reported in our ancillary analyses, resulted from the comparison of the cost of locomotion actually measured with that calculated based upon the multivariable regression model, which is a methodological limitation of the study. Fourth, as participants performed the 6 mWT over ground at their individually selected maximal speed, we were able to assess only one point of the U-shaped speed-metabolic demand curve and we had to assume that participants spontaneously adopted a speed of walking at or near the speed at which energy expenditure is minimized.

In conclusion, although our findings should be taken with caution, nevertheless the message to take home from this study is that lower limb length actually affects the energy cost of overground walking and that small changes in lower limb length might substantially affect walking economy. In operational terms, this means that the energy cost of overground walking might be reduced by simply “manipulating” lower limb length (e.g., by using assistive devices/orthosis, such as slightly thicker shoe soles and insoles, properly manufactured to preserve the physiological function of the ankle-foot complex), thus overcoming, at least in part, the limitation to physical performance imposed by the cardiopulmonary system. The clinical relevance of these findings is that such a relatively small reduction in the energy cost of walking has the potential to dramatically improve mobility in older persons, or in patients with severely limited cardiopulmonary capacity, and, consequently, to improve their independence in the activities of daily living and their quality of life, which is an important target for cardiopulmonary and geriatric rehabilitation.

However, future studies are needed to confirm our findings and to verify, through an appropriate study design, whether experimentally induced increases in lower limb length actually reduce the energy cost of overground walking in older persons.

## Figures and Tables

**Table 1:** General characteristics of the study sample and results from the six-minute walk test (*n* = 62).

General characteristics	
Age, years (mean ± SD)	69.1 ± 5.4
Female sex, *n* (%)	12 (19)
Weight, kg (mean ± SD)	72.3 ± 13.0
Height, cm (mean ± SD)	169.8 ± 7.7
Lower limb length, cm (mean ± SD)	87.7 ± 5.6
Lower limb length to height ratio, % (mean ± SD)	51.7 ± 1.9
Results from the six-minute walk test	
Distance walked, m (mean ± SD)	424 ± 63
Mean walking speed, m sec^−1^ (mean ± SD)	1.2 ± 0.2
Mean kinetic energy, joule (mean ± SD)	51.6 ± 18.4
Step number, *n* (mean ± SD)	310 ± 42
Step cadence, sec^−1^ (mean ± SD)	0.86 ± 0.12
Step length, m (mean ± SD)	1.38 ± 0.19
Step number per meter, m^−1^ (mean ± SD)	0.74 ± 0.10
Resting metabolic rate, joule min^−1^ (mean ± SD)	5,312 ± 958
Gross energy expenditure throughout the test, joule (mean ± SD)	129,362 ± 30,213
Net energy expenditure throughout the test, joule (mean ± SD)	97,489 ± 25,617
Cost of locomotion, joule kg^−1^ sec^−1^ (mean ± SD)	3.73 ± 0.64
Cost of locomotion to kinetic energy ratio, kg^−1^ sec^−1^ (mean ± SD)	79.9 ∗ 10^−3^ ± 26.5 ∗ 10^−3^

**Table 2:** Multivariable regression model testing the association of the energy cost of overground walking with anthropometric measures.

Model: Obs = 62; *F* = 12.75; Prob > *F* < 0.001; adjusted *R* ^2^ = 0.278.			
Cost of locomotion to kinetic energy ratio (kg^−1^ sec^−1^)	*β* ± SE (*β*)	*P*	Standardized *β*
Lower limb length (cm)	−3.72 ∗ 10^−3^ ± 0.74 ∗ 10^−3^	<0.001	−0.78
Lower limb length to height ratio (%)	6.61 ∗ 10^−3^ ± 2.14 ∗ 10^−3^	0.003	0.48

*β* = *β*-coefficient (slope); SE (*β*): standard error of the *β*-coefficient; standardized *β* = *β*-coefficient according to the *Z*-score calculated after transforming all variables as having a mean of 0 and an SD of 1. The *α*-constant (intercept) was 64.89 ∗ 10^−3^.

The initial model also included age and sex that were removed by backward selection (*P* = 0.170 and 0.188, resp.).
